# Development of fuel cell power technology for charging high-temperature superconducting coil: Effect of variable resistor

**DOI:** 10.1016/j.heliyon.2025.e42560

**Published:** 2025-02-12

**Authors:** Young Min Seo, Hyun Woo Noh, Tae Hyung Koo, Rock Kil Ko, Dong Woo Ha

**Affiliations:** Hydrogen Electric Research Team, Korea Electrotechnology Research Institute, Changwon 51543, Republic of Korea

**Keywords:** Fuel cell, Power technology, Superconducting coil, Current charging, Variable resistor

## Abstract

This study focuses on developing fuel cell power technology for charging superconducting coils using variable resistor. For this purpose, a variable resistor is fabricated, and experimental equipment is built to control the amount of current applied to the superconducting coil by fixing the amount of gas supplied to the fuel cell and adjusting its driving voltage. The current and magnetic flux densities increase and decrease depending on the variable resistor. A variable resistor is modified by considering the characteristic curve of the fuel cell, and the flow rate supplied to the unit cell is controlled to overcome inefficient energy use. The amount of current charged to the superconducting coil increases depending on the supplied flow rate. Therefore, the measured magnetic flux density increases according to the charged current. Consequently, the fuel cell characteristic evaluation results and data measured while driving the superconducting coil are almost identical.

## 1 Introduction

A fuel cell is an electrochemical energy source that converts the chemical energy of a fuel into electricity through a redox reaction. The voltage of a unit cell is typically close to 1 V in open-circuit conditions and approximately 0.6 V under nominal power generation conditions. In most practical applications, power management can be performed using electronic converters, especially to increase the voltage to typical application levels, because fuel cells are inherently low-voltage sources [[1], [2], [3]]. However, superconducting coils require an extremely low voltage and a high DC current supply, and the current ripple of the power source must be low. Therefore, fuel cells can be directly applied as a DC power source. Furthermore, fuel cells have the advantage of being able to function as both self-generation and precision power sources, even in environments where the current supply is difficult, such as when power is not supplied or in a moving situation [[4], [5], [6], [7]].

The potential of fuel cells to control current through low voltage and hydrogen flow rate can be exploited; in particular, superconducting coils are well suited to the slow power characteristics of polymer electrolyte membrane fuel cells (PEMFCs). It is preferrable to have the slowest fluctuations possible because losses in the superconducting coils and wires occur for variable currents. In the case of superconducting coils that require high current conduction, the fuel cell current can be increased by increasing the active area of the unit cell or connecting a sufficient number of unit cells in parallel to reach the required current. Moreover, this can be accomplished by placing additional PEMFCs in series to increase the speed of the current increase. These fuel cell characteristics enable them to be used as power sources for superconducting coils [[8], [9], [10], [11], [12]].

A variable resistor has an electrical resistance that can be adjusted. In essence, a variable resistor is an electromechanical transducer that normally works by sliding a contact (wiper) over a resistive element. The advantage of variable resistors is that the voltage is better controlled and the amount of voltage flowing through a circuit can be adjusted. The disadvantage of variable resistors is that they are necessary in specific locations, which would require more parts if one wanted to separate the circuit into different parts. In addition, variable resistors do not work in any area where vibration is involved. Variable resistors can be found in many applications in daily life, such as radios, light dimmers, fan speed controllers, light bulbs, and street lighting. Therefore, several studies have been conducted to improve the performance of various fuel cell systems using variable resistors [[13], [14], [15], [16], [17], [18], [19], [20]].

Bétournay et al. [13] investigated the effects of underground mining conditions, gases, dust, shock, and vibrations on the performances of PEMFCs. The variable resistor was adjusted to give preselected milliampere values. As a result, neither the voltage–amperage curve nor the power–amperage curve showed significant damage effects, and a posttesting stack inspection showed a minor pressure drop at the higher current density and airflow rate. Durr et al. [14] reviewed existing dynamic electrical battery models and described a new mathematical model for a lead acid battery using a nonlinear function for the maximum available energy related to the battery discharge rate. The variable resistor was adjusted depending on the state of charge, and an electrical model was developed with a temperature compensation element to model the different rates of charge and discharge.

Weinmueller et al. [15] investigated the concept of a flexible direct methanol micro fuel cell (FDMMFC) based on the Cr/Au microstructure. The electrical characterization of the FDMMFC was analyzed using a variable resistor simulating an electrical load as well as a classical galvanostatic measurement technique. Dewan et al. [16] developed a method of testing microbial fuel cell (MFC) performance based on storing energy in a capacitor. When a capacitor is connected to an MFC, it acts as a variable resistor and stores energy from the MFC at a variable rate. Thus, the MFC tester can be used to evaluate and optimize energy harvesting when an MFC is used with a capacitor to power wireless sensors to monitor the environment.

Duran et al. [17] proposed a flexible DC load to test and evaluate the current–voltage characteristics of fuel cell stacks and photovoltaic modules based on DC–DC converters. A variable resistor was used to measure the current–voltage characteristic curves of the photovoltaic and fuel cell sources. Consequently, the proposed DC load could emulate the desired load profile with a high degree of accuracy and without any considerable delay. Kim and Chang [18] addressed the issue of voltage reversal (VR), which occurs frequently in stacked MFC systems. The entire internal resistance of the circuit was manipulated by inserting a variable resistor that acted as an ohmic resistance inside the MFC system. As a result, VR was eliminated by controlling the inner current in the parallel-connected cells by manipulating the internal resistance through ohmic resistance tuning.

Baum et al. [19] presented an electrical equivalent circuit model for PEMFC stacks representing the static and dynamic electrical behaviors of a fuel cell stack under pulsed loads. The output voltage of the cell was modeled to represent the different overvoltage mechanisms using current-controlled variable resistor. Consequently, the time-domain simulated behavior of the parameterized model accurately represented the measured behavior. Nurdin et al. [20] derived a theoretical expression for the efficiency of a fuel cell system comprising a proton-exchange membrane fuel cell stack coupled at its output to a DC/DC boost converter. The output terminal of the converter was attached to an electrical load that was modeled as a variable resistor. The results provided strong theoretical and experimental evidence for a unique maximum efficiency point for the fuel cell system.

Most studies have used variable resistors to simulate electrical loads on the electrical characteristics of fuel cells or measured current–voltage characteristic curves by adjusting the overall internal resistance of the circuit. However, few researchers have analyzed the effect of variable resistor in relation to the development of fuel cell power technology for charging superconducting coils. Although the low-voltage and high-current generation characteristics of a fuel cell match well with the zero-electrical-resistance characteristics of superconductivity, the rise rate of the current must be adjustable, similar to a general power source. Superconducting coils require a very low voltage and a high DC supply, and the current ripple of the power source must be low. Because of the characteristics of superconducting devices with zero electrical resistance, a small number of stacks is used, which can significantly reduce the price incurred by fuel cell utilization. Therefore, in this study, the focus was on developing a fuel cell power technology for charging superconducting coils using variable resistor. A variable resistor was fabricated, and the experimental equipment was built. In addition, the current stability was analyzed when a variable resistor was used, and the current characteristics were investigated as a function of the gas flow rate at the desired current application rate by controlling the flow rates of hydrogen and oxygen. The results of the fuel cell characteristics and superconducting coils were compared based on these results.

## 2 General model of voltage loss for fuel cell

A fuel cell is a device that converts chemical energy into electrical energy, and the chemical energy is expressed as the Gibbs free energy. The Gibbs free energy changes depending on the temperature, pressure, and concentration of the reaction gas. Therefore, the theoretical maximum power for the open-circuit voltage (OCV) obtained for the fuel cell is 1.229 V. The well-known empirical equation expresses the voltage drop resulting from the activation loss, ohmic loss, and mass transfer loss at a specific starting point, and it is usually expressed in the following form:

(1) $$V=E_{Nernst}-Losses$$

The Nernst potential ($E_{Nernst}$) is the OCV, the value of which depends on the type of fuel and oxidizer, concentration of each species, operating temperature, operating pressure, etc. The Nernst model can be obtained by deriving the change in the Gibbs free energy, which is expressed in Eq. (2).

(2) $$E_{Nernst}=E_{o}-\frac{R\cdot T}{n\cdot F}\left[\ln\;\left(\frac{P_{H_{2}O}\cdot P_{O_{2}}^{0.5}}{P_{H_{2}O}}\right)\right]$$

Here, $E_{o}$ is the equilibrium electrode potential, R is the gas constant (8.3144 J/mol K), T is the absolute temperature, F is the Faraday constant (96,495 C/mol), and P is the partial pressure.

The output voltage of the actual fuel cell stack is always lower than the OCV because of voltage loss. This voltage loss is called “polarization” and can be broadly divided into activation polarization, resistance polarization, and concentration polarization.

Activation polarization is an overvoltage phenomenon required to overcome the activation energy of the electrochemical reaction that occurs on the catalyst surface. It can also be interpreted as an electrochemical process induced by an applied overvoltage to promote a reaction that can bring an electrochemical cell out of equilibrium as an electric current flows through the cell. The regional resistance of the anode and cathode and the corresponding activation loss $\eta_{Act}$ are expressed as [[21], [22], [23], [24]]

(3) $$\eta_{Act,j}=\frac{R\cdot T}{\alpha \cdot n\cdot F}\ln\left(\frac{i}{i_{O,j}}\right),j=anode,cathod$$

(4) $$\eta_{Act}=\eta_{Act,an}+\eta_{Act,cat}$$

where α is the transition coefficient, n is the coefficient of electrons participating in the reaction, $i_{O}$ is the exchange current density, and i is the current density.

In a fuel cell, resistance exists when electrons flow from the fuel electrode to the air electrode and ions flow through the electrolyte. This phenomenon is called “ohmic polarization,” and the resulting voltage loss is called “ohmic loss.” Its size is based on Ohm's law. The total resistance is defined as the sum of the anodic, cathodic, and electrolytic ohmic losses, and its reaction equation is [[25], [26], [27], [28]].

(5) $$\eta_{Ohm,j}=i\cdot r_{j},j=anode,cathode$$

(6) $$\eta_{Ohm}=\eta_{Ohm,an}+\eta_{Ohm,el}+\eta_{Ohm,cat}$$

Concentration polarization occurs because an insufficient mass transfer occurs owing to the depletion of reactants or the accumulation of products in the boundary layer close to the electrode catalyst surface. Concentration polarization can be minimized if the flow of the reactants and products is controlled by optimizing the electrode and flow structure of the fuel cell. The concentration polarization varies depending on the type of gas, thickness of the electrode, and porosity and tortuosity of the electrode. The concentration loss from the sum of the two anodic and cathodic concentration losses is [[29], [30], [31], [32]].

(7) $$\eta_{Conc,an}=-\frac{R\cdot T}{2\cdot F}\ln\left(1-\frac{i}{i_{l,an}}\right)+\frac{R\cdot T}{2\cdot F}\ln\left(1+\frac{P_{H_{2}O}\;\cdot \;i}{P_{H_{2}O}\;\cdot \;i_{l,an}}\right)$$

(8) $$\eta_{Conc,cat}=\frac{R\cdot T}{2\cdot F}\ln\left(1-\frac{i}{i_{l,cat}}\right)$$

(9) $$\eta_{Conc,}=\eta_{Conc,an}+\eta_{Conc,cat}$$

where $i_{l,an}$ and $i_{l,cat}$ represent the limiting current densities of the anodic and cathodic electrodes, respectively.

As mentioned previously, various voltage losses cause decreases in the performance and lifespan of fuel cells. To maximize the efficiency and durability of fuel cells, factors causing voltage loss must be accurately measured and analyzed. In addition, because superconducting coils require an extremely low voltage and high DC supply, as well as a low current ripple of the power source, a fuel cell unit cell can be used directly as a DC power source. Because of the characteristics of superconducting devices with an electrical resistance of zero, high voltage is not required. Consequently, a fuel cell stack with a small number of stacks is used, which can significantly reduce the price incurred because of fuel cell utilization. Therefore, through technological convergence, the technical shortcomings of fuel cells and superconductivity are complemented, and their advantages are maintained.

**Fig. 1:**
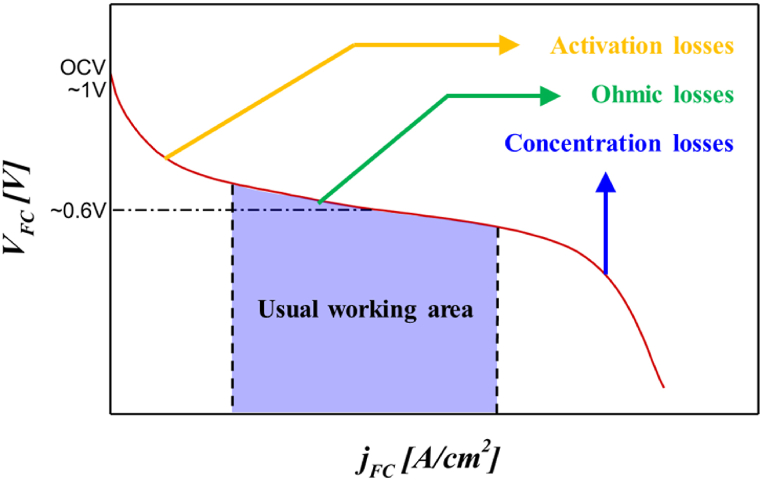
*I–V* curve for unit cell of fuel cell.

Fig. 1 shows the *I–V* curve of a typical fuel cell unit cell. Generally, the driving voltage of a fuel cell is 0.6 V under nominal power generation conditions, and, as the voltage decreases, the current increases. The characteristic curve of the fuel cell is determined based on the unique performance of the unit cell and the amount of gas injected into it. Once the characteristic curvature of the fuel cell is determined, the driving voltage can be changed by changing the resistance of the circuit. Fuel cells and superconducting coils share remarkably similar characteristics. In the case of an ideal superconductor, the resistance is zero, allowing a large current to flow at a very low voltage (close to 0 V). Similarly, fuel cells can produce a current exceeding 1 A/cm^2^, depending on the membrane electrode assembly (MEA) area, within an operating voltage range of 0.4–0.7 V per unit cell. This indicates that superconducting coils can be charged using fuel cells. Because the resistance of the superconducting magnet in liquid nitrogen is zero, if there is no resistance in the circuit, the unit cell operates at less than 0.2 V. Based on these principles, a fuel cell power technology was developed in this study by charging a superconducting coil using a variable resistor.

## 3 Superconducting coil experiment

### 3.1 Fabrication of variable resistor

Superconducting wires can conduct higher currents than copper wires because of the nature of superconductivity, in which the resistance becomes zero below the critical temperature. In the case of power sources used in general wires, a high voltage and low current are usually required. For this reason, most power supplies are fabricated with a high voltage and low current. For power sources used in superconducting wires, however, a power supply that can conduct higher currents is required, and power devices dedicated to superconductivity are fabricated using a special power supply with a low voltage and high current.

Proton exchange membrane fuel cells (PEMFCs) produce electricity using hydrogen gas and oxygen gas, which is an eco-friendly material. Generally, a unit cell consisting of a membrane electrode assembly (MEA) has an operating voltage of 0.4–0.8 V. The current is determined by the MEA surface area and has a performance of approximately 1.0 A/cm^2^. In general, devices using PEMFCs require high voltage; therefore, a stack of multiple MEAs is usually employed. However, in the case of superconductivity, it is possible to operate sufficiently with the driving voltage of a unit cell fabricated from a single MEA.

When driving a superconducting coil using a unit cell, the easiest way to control the current is to control the amounts of hydrogen and oxygen gas supplied to the unit cell. However, when the amount of current supplied to the superconducting coil is controlled by adjusting the amounts of hydrogen and oxygen gas, the control time of the current is delayed by the time required for the chemical reaction of the gas. To compensate for this shortcoming and control the current quickly, a variable resistor was fabricated to control the amount of current applied to the superconducting coil by fixing the amount of gas supplied to the fuel cell and adjusting the fuel cell driving voltage.

**Fig. 2:**
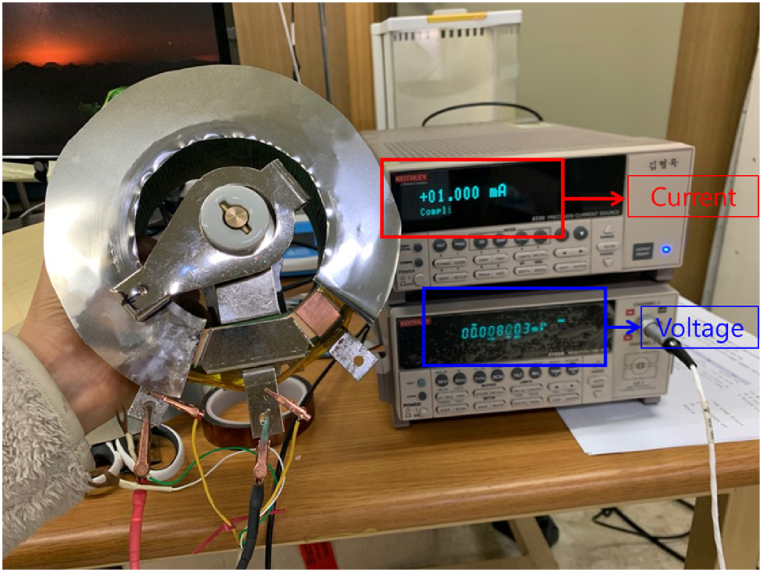
Measurement for variable resistor to change the driving voltage of the unit cell.

Fig. 2 shows the variable resistor fabricated to change the driving voltage used in this study. After the variable resistor was fabricated, the resistance at each location in the variable resistor was measured using a nanovoltmeter and current source equipment. The resistance of the variable resistor was determined by referring to the driving voltage data of the unit cell according to the resistance. The variable resistor fabricated in this study stably adjusted the resistance without noise when its shaft was rotated. The fabricated variable resistor can be adjusted from a maximum of 0.06 Ω to a minimum of 0.004 Ω according to resistor distance. Therefore, current regulation from approximately 10 to 25 A is possible using a variable resistor. Table 1 lists the driving voltage and the amount of current applied to the coil according to the resistance.

**Table 1:** Driving voltage of unit cell and current amount applied to coil.

Resistor distance [cm]	Resistance [Ω]	Unit cell voltage [V]	HTS coil current [A]
15	0.06	0.77	10.8
10	0.04	0.76	12
6	0.024	0.735	14.2
4	0.016	0.715	17
2	0.008	0.65	21
1	0.004	0.62	23

A variable resistor typically uses three terminals, and the desired resistance value is obtained by rotating the central shaft or pushing or pulling the handle back and forth. In this study, a method of rotating the central shaft was considered. A rotary variable resistor operates through a movable wiper that glides over a circular resistive element. The two ends of the resistive element are connected to fixed terminals, while the wiper is connected to a central terminal. As the central shaft rotates, the wiper adjusts its position along the resistive element, thereby varying the resistance in the circuit. This mechanism is designed to maintain electrical continuity within a defined range of movement, ensuring stability and enabling either linear or nonlinear resistance changes. However, allowing 360-degree rotation poses challenges; the wiper may move beyond the resistive element's endpoints, resulting in an open circuit or, in the worst case, a short circuit if it contacts both endpoints simultaneously. To mitigate these issues, most rotary variable resistors incorporate mechanical stoppers to limit the rotation angle, reducing wear and tear and enhancing durability and reliability. Accordingly, in this study, repeated experiments were conducted manually by rotating the resistor at a consistent speed as closely as possible, without motorized control. In repeated experiments, values of 10, 15, and 20 A were measured at the specified positions, depending on the degree to which the shaft was rotated. Based on this variable resistor, the characteristics of the superconducting coil were evaluated by adjusting the resistance of the entire circuit of the drive device of the fuel cell constructed.

### 3.2 Current stability test

**Fig. 3:**
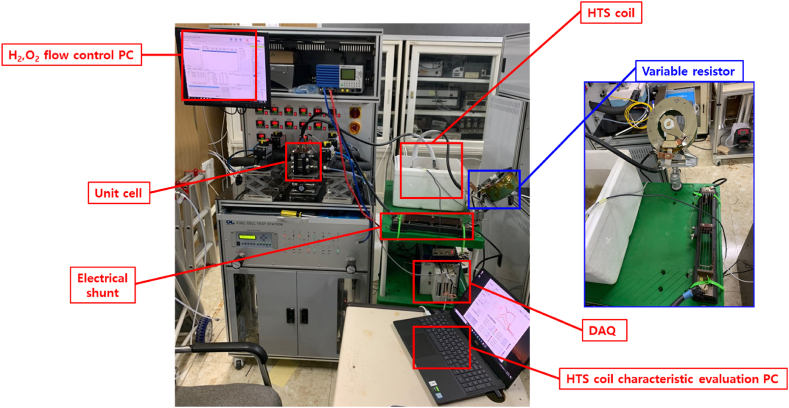
Equipment configured for continuous current measurement using a variable resistor.

Fig. 3 shows the equipment configured for current measurement using the variable resistor. The experimental equipment considered can be roughly classified into seven types: the hydrogen and oxygen flow control PC, fuel cell unit cell, electric shunt, high-temperature superconducting (HTS) coil, variable resistor, data acquisition (DAQ) system, and HTS coil characteristic evaluation PC. The following roles were assigned to the components.

· The hydrogen–oxygen flow control PC controls the amounts of hydrogen and oxygen supplied to the polymer electrolyte membrane unit cell and collects data, such as the temperature, voltage, and current of the unit cell.
·The unit cell produces the current for driving the superconducting coils.
·The electric shunt is used to check the current at which the superconducting coil is charged.
·The superconducting coil generates a magnetic field using the current produced by the fuel cell.
·The variable resistor controls the current applied to the superconducting coil by adjusting the driving voltage.
·The DAQ system receives the superconducting magnetic field measurements and shunt data values.
·The HTS coil characteristics evaluation PC is used to plot and save the data received in the DAQ system.

**Fig. 4:**
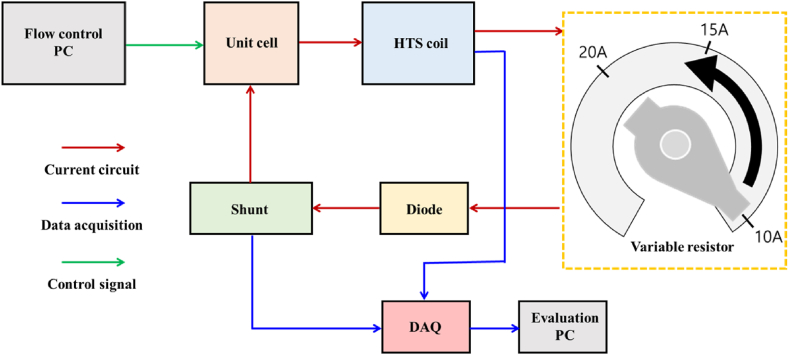
Schematic diagram for continuous current measurement using a variable resistor.

Fig. 4 shows the schematic diagram for continuous current measurement using a variable resistor. In this study, the superconducting coil was charged using a variable resistor. The variable resistor is part of the current circuit in the fuel cell power supply for charging the superconducting coil. A variable resistor was added to the circuit connecting the fuel cell unit cell and the superconducting coil, and an electrical shunt was added to check the amount of current with which the superconducting coil was charged. When the amount of current charged to the superconducting coil was controlled using a variable resistor, the amounts of hydrogen gas and oxygen gas supplied to the unit cell according to the control signal line were fixed at 1 and 2 L/min, respectively. To determine whether the superconducting coil operated normally, a magnetic field sensor was attached to the center of the HTS coil, and the magnetic field value was measured according to the current. All data were collected using the DAQ equipment and controlled using the LabView program installed on the operating PC according to the data acquisition line.

The primary advantages of charging superconducting coils using fuel cells and variable resistors are efficiency and controllability. Fuel cells efficiently convert chemical energy into electrical energy, providing stable and continuous power, which makes them particularly suitable for high-efficiency energy systems such as superconducting coils. Additionally, variable resistors enable precise control of the current during the charging process, preventing overcurrent or overheating during the magnetic field generation of the superconducting coil. This enhances the safety and reliability of the system. Together, this combination allows for stable and efficient superconducting coil charging while minimizing energy loss.

**Fig. 5:**
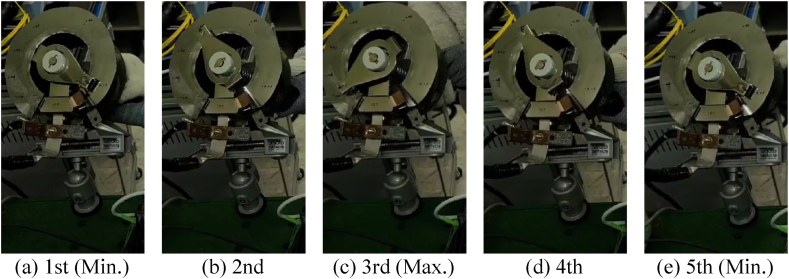
Continuous changes or fixed values of variable resistor from maximum resistance to minimum resistance.

Fig. 5 shows an experimental test for the continuous changes or the fixed values from maximum to minimum resistance. First, the values of the voltage measured in the coil and fuel cell and the current and magnetic flux density in the coil were measured while continuously moving the shaft of the variable resistor in counter-clockwise and clockwise directions for approximately 70 s. The variable resistor could not rotate 360° because of structural reasons in the lower area, and its measurements were made in the remaining areas, except at approximately 45° in the lower area. In this study, repeated experiments were performed to obtain results without the noise caused by manual work by rotating at as close to the same speed as possible. Second, the other experiment was conducted to determine whether the amount of current applied to the superconducting coil was stable when the resistance was fixed. In advance, values of 10, 15, and 20 A were indicated according to the degree to which the shaft of the variable resistor rotated. The values of the voltage measured in the coil and fuel cell and the current and magnetic flux density in the coil were measured according to the fixed value of the resistance.

**Fig. 6:**
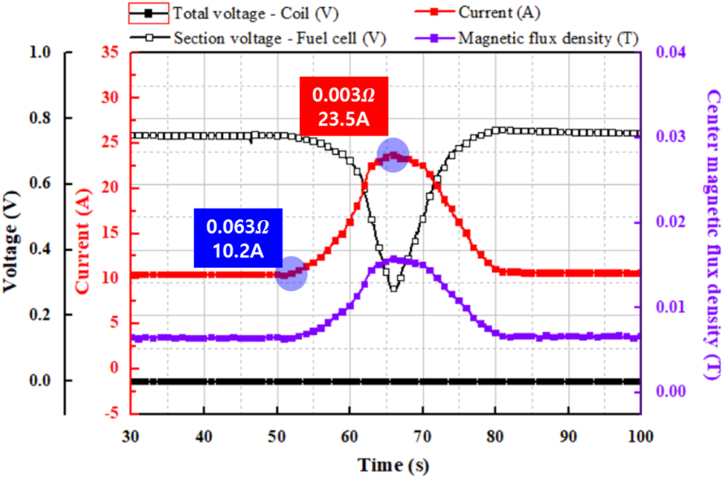
Measurement results for continuous change of variable resistor.

Fig. 6 shows the measurement results for the continuous change in the variable resistor, including such results as the magnetic field value depending on the amount of current charged to the HTS coil. The voltage presented in the graph consists of the operating voltage of the fuel cell and the voltage across the superconducting coil. When the superconducting state is maintained, the resistance of the superconducting coil approaches zero, resulting in a voltage value that is nearly 0 V. Conversely, if the superconducting state is disrupted, the resistance of the coil increases sharply, leading to a rapid increase in voltage. When the resistance of the variable resistor was 0.063 Ω, the superconducting coil was charged to its minimum value of approximately 10.2 A. In addition, when the driving voltage was lowered by lowering the value of the resistance, the superconducting coil was charged to the maximum value of approximately 23.5 A at 0.003 Ω. The magnetic field generated by the superconducting coil is proportional

to the current. The strength of the magnetic field depends on both the number of turns (*N*) of the coil and the current (*I*). Since the same superconducting coil is used, the magnetic field strength increases proportionally with the current. As a result, the magnetic flux density of the superconducting coil increased, and then it decreased as the variable resistor shaft rotated continuously, depending on the variation of current. Consequently, the voltage of the fuel cell decreased and then increased, whereas that of the superconducting coil remained unchanged. In addition, while the amount of current charged to the superconducting coil was controlled, there was no significant noise, and the response was fast.

**Fig. 7:**
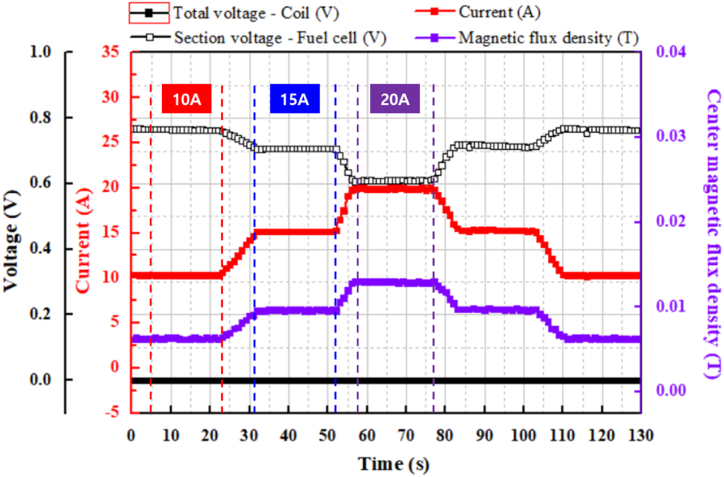
Measurement results for the fixed value of variable resistance.

Fig. 7 shows the stability of the current applied to the superconducting coil according to the fixed resistance value. When the flow rates of hydrogen and oxygen supplied to the unit cell are constant, the performance of the unit cell is determined from the *I–V* curve. It is important to check whether the amount of current applied to the superconducting coil is stable while the driving voltage is maintained at a certain point on the *I–V* curve according to the resistance value. The experiment was repeated by adjusting the resistance value for approximately 130 s and maintaining the current for 20 s in the sections with values of 10, 15, and 20 A. The current and magnetic flux density increased and decreased in a stepwise manner depending on the fixed value of the resistance. Consequently, the voltage of the fuel cell decreased and increased in steps, but that of the superconducting coil showed no change. As a result, the desired current was maintained stably without noise.

**Fig. 8:**
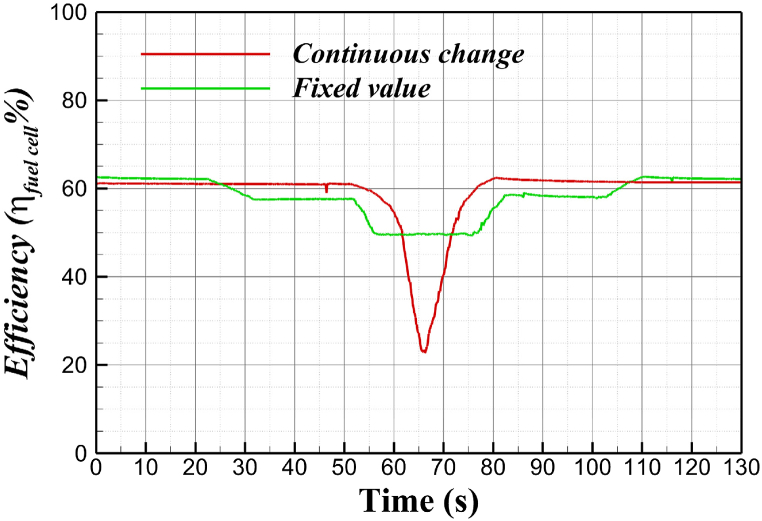
Efficiency of fuel cell for variable resistor.

Fig. 8 shows the efficiency of fuel cell for variable resistor. The efficiency of a fuel cell is generally defined as follows:

(10) $$\eta_{fuel\;cell}=\frac{E_{H2}}{{\Delta \bar{h}}_{f,LHV}}$$

Here, E represents the electrical energy generated from 1 mol of fuel, and ${\Delta \bar{h}}_{f,LHV}$ is the formation enthalpy. Assuming that all the energy generated by the hydrogen fuel cell, including the formation enthalpy, is converted into electrical energy, the electromotive force is approximately 1.25 V (based on the lower heating value, LHV). Considering the fuel utilization rate, this value can be approximated to 1.23 V. By dividing this value by the operating voltage of the cell, the voltage efficiency of the cell can be determined.

The voltage of the stack was measured by continuously adjusting the variable resistor to monitor the current applied to the coil and the driving limit of the fuel cell, as shown in Fig. 6. The current supplied to the fuel cell increased gradually from 10 A to 23 A as the variable resistor was adjusted, while the efficiency decreased from approximately 61 %–23 %. Especially, when 20 A of current was applied to the cell, the cell voltage was about 0.6 V. Beyond this operating point, the concentration loss increased rapidly, causing a steep drop in voltage. To prevent damage to the cell, the maximum applied current was limited to 20 A. Additionally, the current was varied in a stepwise manner, and the voltage of the stack was measured to assess whether the current applied to the coil could be maintained continuously while preserving the superconducting state, as shown in Fig. 7. The fuel cell efficiency was tested stepwise at 10 A, 15 A, and 20 A applied to the coil, with efficiencies of 62 %, 58 %, and 50 %, respectively.

When charging a superconducting coil using a fuel cell and a variable resistor, the efficiency of the fuel cell can vary depending on the supplied current. Generally, the efficiency of the fuel cell is relatively high at low current levels; however, as the current required by the superconducting coil increases, the efficiency tends to decrease. This decline is due to energy losses that occur when the fuel cell supplies current, and power consumption rises as the current increases. Additionally, if the operating voltage of the fuel cell is adjusted using a variable resistor, a constant voltage may be supplied to the superconducting coil, leading to changes in efficiency. Therefore, to optimize fuel cell efficiency when charging a superconducting coil, it is important to carefully analyze the relationship between efficiency and current, taking into account the required current range.

### 3.3 Gas flow effect

**Fig. 9:**
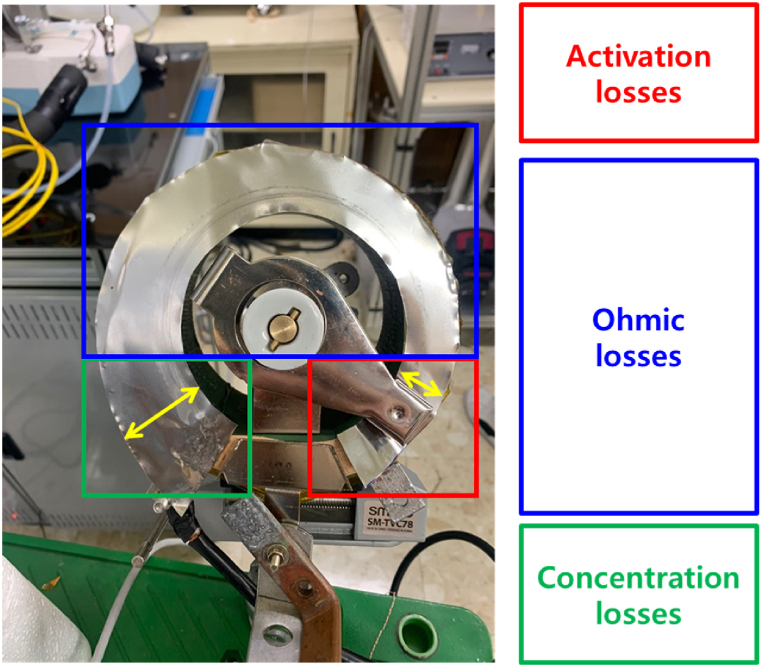
Manufacturing of variable resistor considering the characteristic curve of the fuel cell.

Fig. 9 shows a variable resistor modified by considering the characteristic curve of the fuel cell. An ideal fuel cell should maintain a constant voltage, which is thermodynamically defined, but the actual voltage output is lower than the expected low pressure owing to the losses in each process. In the *I–V* curve for the performance of the fuel cell, the loss increases and the voltage decreases as the current density increases. In particular, the ohmic polarization region, which is the working region of the fuel cell, is distributed in the intermediate current density region, and the voltage decreases linearly as the current density increases. However, the activation polarization and concentration polarization regions change significantly as the current density increases. Therefore, to prevent sudden changes caused by manual work, the range of resistance fluctuation was reduced as the voltage was lowered or the current was increased. As shown in Fig. 9, it was fabricated such that the thickness increased as the shaft of the variable resistor was rotated counterclockwise, considering the characteristic curve of the fuel cell. As mentioned previously, fixing the gas flow rate and controlling the amount of current charged to the superconducting coil using a variable resistor has the advantages of quick response and signal stability.

In this study, three main flow rates were supplied to the unit cell. In the first case, both the hydrogen flow rate and the oxygen flow rate were set to 0.5 L/min. In the second case, the hydrogen flow rate was set to 0.5 L/min, and the oxygen flow rate was increased to 1.0 L/min. In the third case, the flow rate of hydrogen gas was increased to 1.0 L/min, and the oxygen flow rate was increased to 2.0 L/min, which is the flow rate that can achieve the maximum performance of the unit cell. By using the same variable resistor, the maximum and minimum resistances were the same for each experiment.

**Fig. 10:**
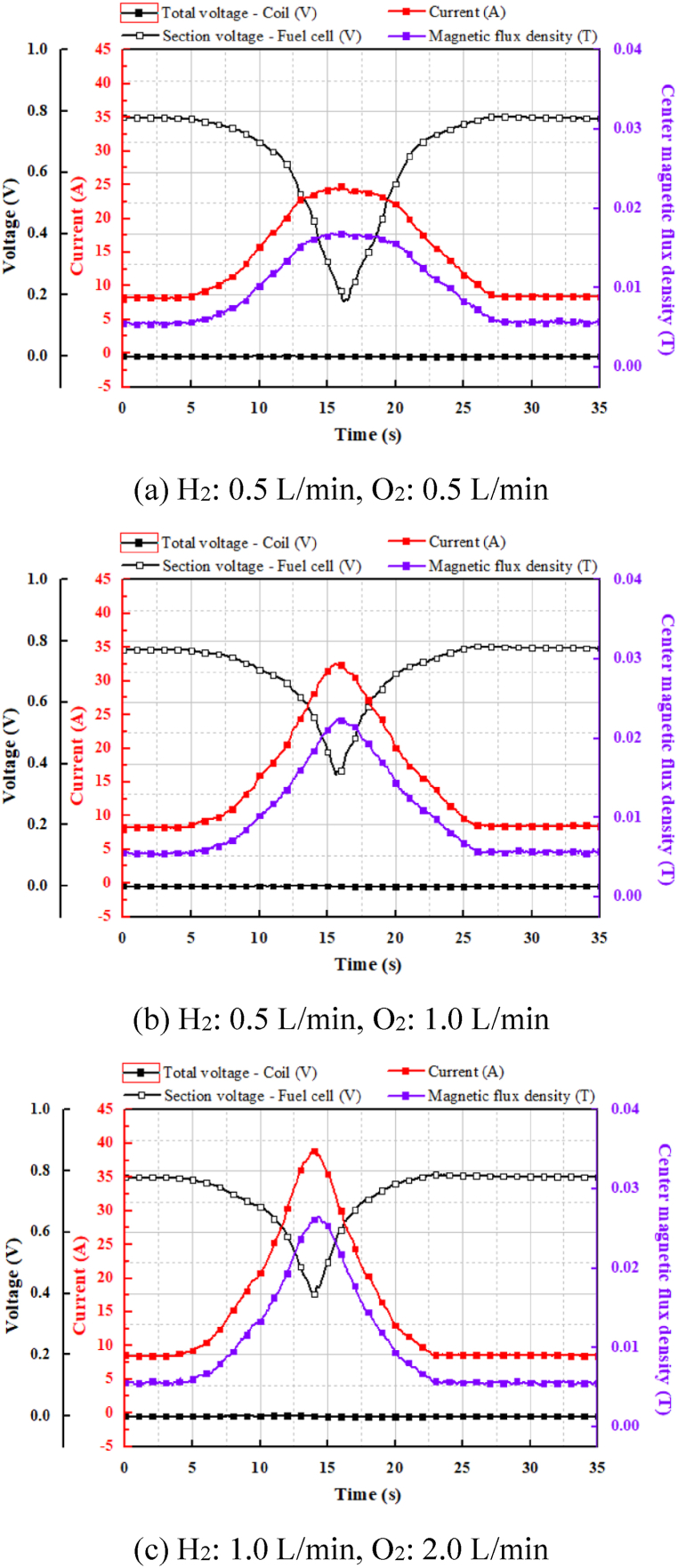
Current characteristics depending on hydrogen and oxygen flow rates.

Fig. 10 shows the characteristics of the current applied to the superconducting coil when the value of the resistance changed depending on the gas flow rate. The overall trend of the measurement data for the modified variable resistor was similar to that for the previous one; however, the amount of current charged to the superconducting coil varied depending on the flow rates of hydrogen and oxygen supplied. In the first case, the minimum value of the current was 8.2 A with the maximum value of the resistance, whereas the maximum value of the current was 23 A with the minimum value of the resistance. In the second case, the minimum current was 8.2 A, and the maximum current was 32.5 A. The flow rate of hydrogen gas was the same

as that in the first case, and the oxygen flow rate was doubled. When the flow rates of the hydrogen gas and oxygen gas were maximized, the minimum current was the same, but the maximum current was charged to approximately 38 A. Therefore, the amount of current charged to the superconducting coil increased depending on the flow rate of the supplied hydrogen and oxygen; consequently, the measured magnetic flux density increased according to the charged current. As a result, the maximum current charged in the superconducting coil for the modified variable resistor increased by approximately 64 % compared with that of the previous one because the thickness increased as the shaft of the variable resistor rotated counterclockwise.

The experimental results show that the maximum current varied significantly depending on the flow rate supplied to the unit cell. When the amount of current charged to the superconducting coil was small, it was advantageous to reduce the flow rate and control the resistance. However, when a high current was required, it was advantageous to control the current by adjusting the value of the resistance while increasing the amount of gas supplied to the fuel cell.

**Fig. 11:**
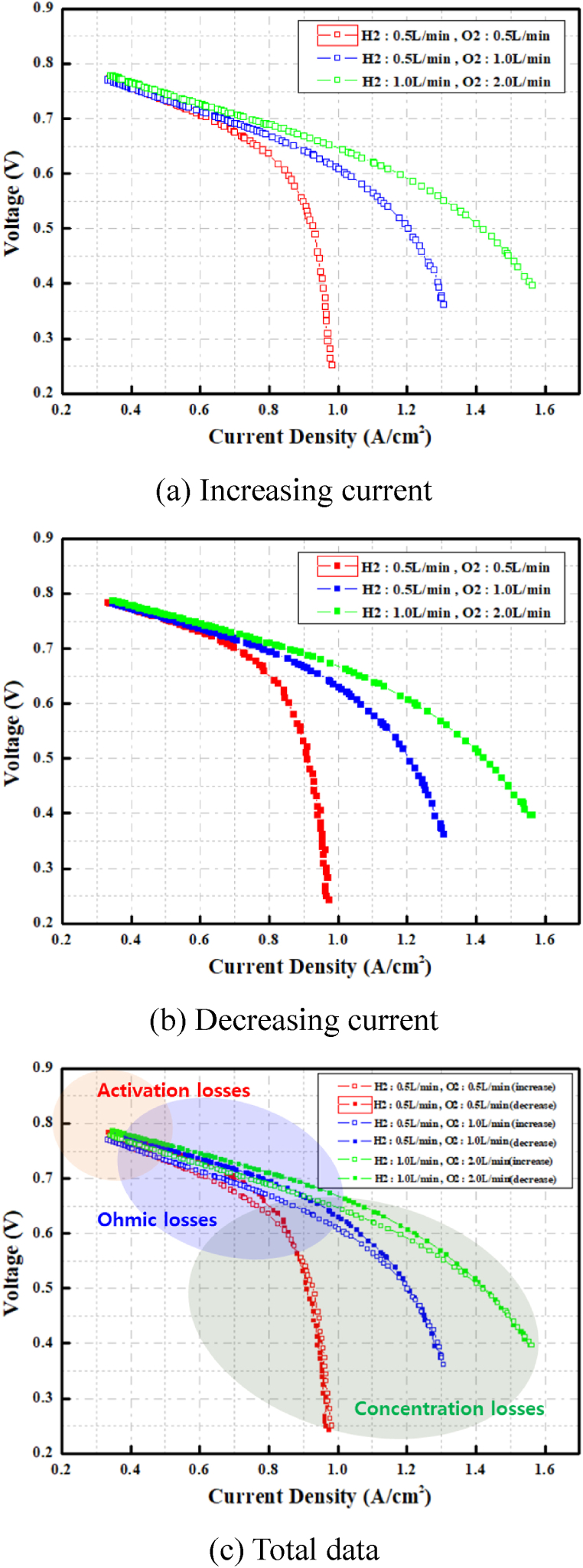
Characteristic curve analysis of unit cell.

Fig. 11 shows the characteristic curve analysis of the unit cell for three flow rates. There is a difference in the characteristic curve when the current density increases and decreases. The voltage when the current density is decreased is slightly higher than that when the current density is increased by the various reasons, such as the electrochemical reactions, material properties, system design, and operating conditions. Although charging and discharging appear to involve reverse processes governed by the same electrochemical reaction, the performance curves differ due to factors such as reaction mechanisms, electrode characteristics, mass transfer, internal resistance, and system configuration. While it is difficult to identify the exact cause, the asymmetry of the electrochemical reaction and the electrical characteristics of the inductance coil within the system significantly influence performance. In addition, the effect

of concentration loss increased in the low-voltage and high-current sections with decreasing flow rate supplied to the unit cell. For this reason, in the three aforementioned cases, the minimum current when adjusting the resistance was the same at 8.2 A, and only the maximum current varied depending on the flow rate.

**Fig. 12:**
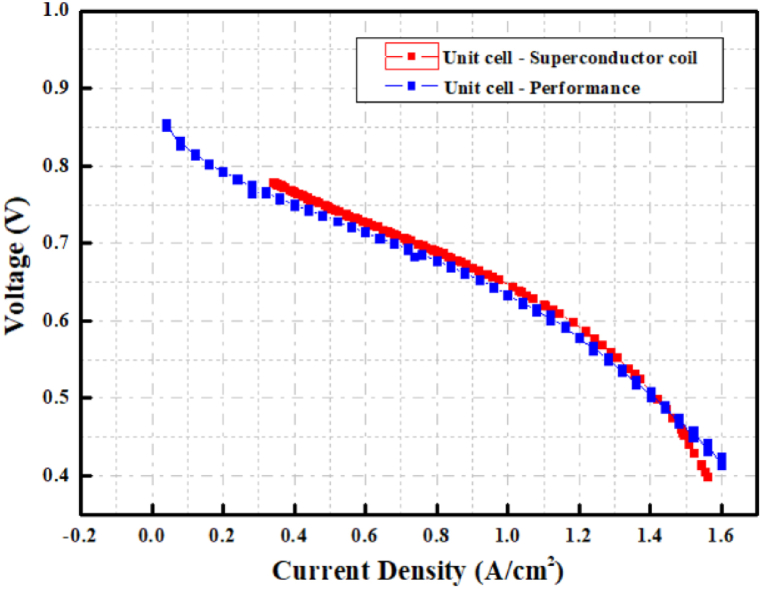
Comparison between fuel cell characteristic evaluation and HTS coil for voltage as a function of current density.

Fig. 12 shows the fuel cell characteristic evaluation data and the results of the voltage–current comparison when driving the superconducting coil. The flow rates were 1.0 L/min for hydrogen gas and 2.0 L/min for oxygen gas, and the current density was 1.12 A/cm^2^. As shown in Eqns. (7)–(9), the concentration loss is caused by the amount of gas supplied to the unit cell of the fuel cell and the amount of gas consumed by the MEA. The fuel cell characteristic evaluation results and the data measured while driving the superconducting coil were almost identical. Therefore, the desired current can be applied to the superconducting coil if the fuel cell characteristics are known.

To conduct an experiment for fuel cell power technology for charging HTS coils, a variable resistor was fabricated, and the current characteristics of the HTS coil were investigated depending on the gas flow rate at the desired current application rate by controlling the flow rates of hydrogen and oxygen. This experimental system can be used to obtain the charging amount of the higher current on the HTS coil depending on various design parameters and operating conditions. Planned future work includes conducting an HTS coil driving experiment by fabricating a unit cell with an MEA with an area of 100 cm^2^, aiming to charge 100-A currents.

## 4 Conclusion

The focus of this study was on developing fuel cell power technology for charging superconducting coils using variable resistor. Based on the fabricated variable resistor and by rotating the central shaft, the characteristics of the superconducting coil were evaluated by adjusting the resistance of the entire circuit of the fuel cell drive device. The values of the voltages measured in the coil and fuel cell, the current, and the magnetic flux density in the coil were measured experimentally with continuous changes and fixed resistance values. The magnetic flux density of the superconducting coil increased, and then it decreased as the variable resistor shaft rotated. In addition, when the amount of current charged to the superconducting coil was controlled, there was no significant noise, and the response was fast.

For the case of continuous changes, the current supplied to the fuel cell increased gradually from 10 A to 23 A as the variable resistor was adjusted, while the efficiency decreased from approximately 61 %–23 %. For the case of the fixed resistance value, the fuel cell efficiency was tested stepwise at 10 A, 15 A, and 20 A applied to the coil, with efficiencies of 62 %, 58 %, and 50 %, respectively. To optimize fuel cell efficiency when charging a superconducting coil, it is important to carefully analyze the relationship between efficiency and current, taking into account the required current range.

A variable resistor was modified by considering the characteristic curve of the fuel cell, and the flow rate supplied to the unit cell was controlled to overcome inefficient energy use. The amount of current charged to the superconducting coil increased depending on the supplied flow rate; consequently, the measured magnetic flux density increased according to the charged current. When a high current is required, it is advantageous to control the current by adjusting the resistance while increasing the amount of gas supplied to the fuel cell. This experimental system can be used to obtain the charging amount of the higher current on the HTS coil depending on various design parameters and operating conditions. Planned future work includes conducting an HTS coil driving experiment by fabricating a unit cell with an MEA with an area of 100 cm^2^, aiming to charge 100-A currents.

## CRediT authorship contribution statement

**Young Min Seo:** Writing – original draft, Visualization, Investigation. **Hyun Woo Noh:** Writing – original draft, Methodology, Conceptualization. **Tae Hyung Koo:** Investigation, Formal analysis, Data curation. **Rock Kil Ko:** Writing – review & editing, Supervision, Methodology. **Dong Woo Ha:** Writing – review & editing, Project administration, Conceptualization.

## Declaration of competing interest

The authors declare that they have no known competing financial interests or personal relationships that could have appeared to influence the work reported in this paper.
